# Genomic profiling of lymph node and distant metastases from papillary and poorly differentiated thyroid carcinomas

**DOI:** 10.1007/s12020-024-03968-0

**Published:** 2024-07-19

**Authors:** Valdemar Máximo, Miguel Melo, Manuel Sobrinho-Simões, Paula Soares, Arnaud Da Cruz Paula

**Affiliations:** 1 i3S – Instituto de Investigação e Inovação em Saúde, Universidade do Porto, Porto, Portugal; 2 IPATIMUP – Instituto de Patologia e Imunologia Molecular da Universidade do Porto, Porto, Portugal; 3 Departamento de Patologia e Oncologia, Faculdade de Medicina, Universidade do Porto, Porto, Portugal; 4 Departamento de Endocrinologia, Diabetes e Metabolismo, Centro Hospitalar e Universitário de Coimbra, Faculdade de Medicina, Universidade de Coimbra, Coimbra, Portugal; 5 Departamento de Patologia, Hospital São João, Porto, Portugal

**Keywords:** Papillary thyroid carcinomas, Poorly differentiated thyroid carcinomas, DNA sequencing, Somatic mutations, Copy number alterations, Distant metastasis

## Abstract

**Purpose:**

To perform a molecular profiling of the metastases from papillary thyroid carcinomas (PTCs) and poorly differentiated thyroid carcinomas (PDTCs).

**Methods:**

We retrieved and analyzed the molecular and clinical features of 136 metastases from PTCs and 35 metastases from PDTCs subjected to targeted DNA sequencing, from cBioPortal. The clinicopathological data included the number and location of the metastases, and genomic data included mutations, translocations, copy number alterations and fraction of the genome altered (FGA).

**Results:**

Bone metastases from PTCs had a lower frequency of *BRAF* mutations than the lymph node metastases (LNMs) (43% vs 88%, *p* < 0.01), and a higher frequency of *RBM10* and *NRAS* mutations than the LNMs (21% vs 3% for both, *p* < 0.05). The FGA of the bone metastases was higher than the FGA of the lung metastases (5.6% vs 1.3%, *p* < 0.05). The frequency of *RET* translocations was higher in the lung metastases from PTCs than the LNMs (15% vs 3%, *p* < 0.05). The LNMs from PTC patients harboring 4 or more distant metastases (DMs) had a higher frequency of *TERT* promoter mutations than the LNMs from patients harboring less than 4 DMs (96% vs 65%, *p* < 0.001). *SDHA* gene amplifications were enriched in the bone metastases from PDTCs and absent in the LNMs (38% vs 0%, *p* < 0.05).

**Conclusion:**

Metastases from PTCs and PDTCs harbor clinically relevant alterations affecting distinct body locations, such as *NRAS* and *RBM10* mutations, *RET* translocations and *SDHA* amplifications that may be explored therapeutically.

## Introduction

Over the past three decades, the incidence of thyroid cancer (TC) has increased and constitutes the most common endocrine malignancy [1]. Considering the major clinical challenges that exist with papillary thyroid carcinomas (PTCs) and poorly differentiated thyroid carcinomas (PDTCs) with distant metastases (DMs), a precise characterization of the genetic profile in these lesions is hence crucial. We have previously retrieved and characterized the genetic repertoire of primary and metastatic TCs, including 276 PTCs and 127 PDTCs subjected to targeted DNA sequencing, from the cBioportal database, and observed a significantly higher frequency of *TERT* promoter mutations, *CDKN2A/B* homozygous deletions and *RET* translocations in the metastasis from PTCs than in primary PTCs [2]. In addition, we have also noticed an enrichment of *TP53* biallelic alterations in the metastases from PDTCs when compared to primary tumors [2]. Within the metastatic setting however, the molecular characterization of the metastases from distinct body locations in patients with PTCs and PDTCs is rather scarce. A recent study has focused on the molecular analysis of 160 PTC patients with lateral neck lymph node metastasis (LNMs), and noticed a high frequency of *BRAF* mutations (68%), followed by *RET* translocations (17%) and *TERT* promoter mutations (5%) [3]. An additional genetic study of 8 PTC patients with symptomatic bone metastases revealed a high frequency of *TERT* promoter mutations (88%) and *RAS* mutations (50%) affecting these patients [4]. Considering such poor knowledge, we aim to characterize the genetic repertoire of the LNMs and DMs from PTC and PDTC patients using a subset of the series we have previously reported [2].

## Materials and methods

### Case selection and massively parallel sequencing analysis

We retrieved the clinicopathological and genomic data of the metastases from PTCs and PDTCs from our previously published study [2]. All of these TCs were subjected to targeted massively parallel sequencing via Memorial Sloan Kettering–Integrated Mutation Profiling of Actionable Cancer Targets (MSK-IMPACT) [5, 6] from 2014 to 2020, which targets all exons and selected introns of 341–468 cancer genes [7]. We retrieved 136 metastases from PTCs including 80 LNMs, 9 head and neck metastases, 33 lung metastases and 14 bone metastases. Due to the same regional occurrences and for practical purposes, the head and neck metastases were included in the group of the LNMs for a total of 89 metastases. We also retrieved 35 metastases from PDTCs, including 20 LNMs (18 LNMs and 2 head and neck metastases), 7 lung metastases and 8 bone metastases. Of note, no synchronous nor multiple metastatic samples per patient were included in this study. The clinicopathological data included age at diagnosis and number and location of the metastases [5]. Relevant genomic data included somatic mutations, structural variants, copy number amplifications and homozygous deletions, tumor mutational burden (TMB) and fraction of the genome altered (FGA) [5, 6]. The copy number segment files were also retrieved to determine whether genes harboring somatic mutations were targeted by loss of heterozygosity [8].

### Genetic comparisons and statistical analysis

The frequencies of genetic alterations were compared between the LNMs and the different DMs from PTC and PDTC patients, and between the number of DMs each PTC and PDTC patient harbored. Regarding the latter, and for increased statistical power, we used a cutoff of 4 DMs to stratify both PTC and PDTC patients. For all of these comparisons, only the 341 genes targeted by the smallest MSK-IMPACT panel were used. Statistical analyses were performed using R v3.1.2. For comparisons between categorical variables, Fisher’s exact tests were employed, whereas for continuous variables, Mann–Whitney U tests were used. All tests were one-sided and a *p*-value < 0.05 was considered statistically significant. For a proper visualization, only recurrently altered genes per TC type are represented in the heatmaps.

## Results

### Diverse genetic profile of the metastases from PTCs according to number and body location

**Fig. 1:**
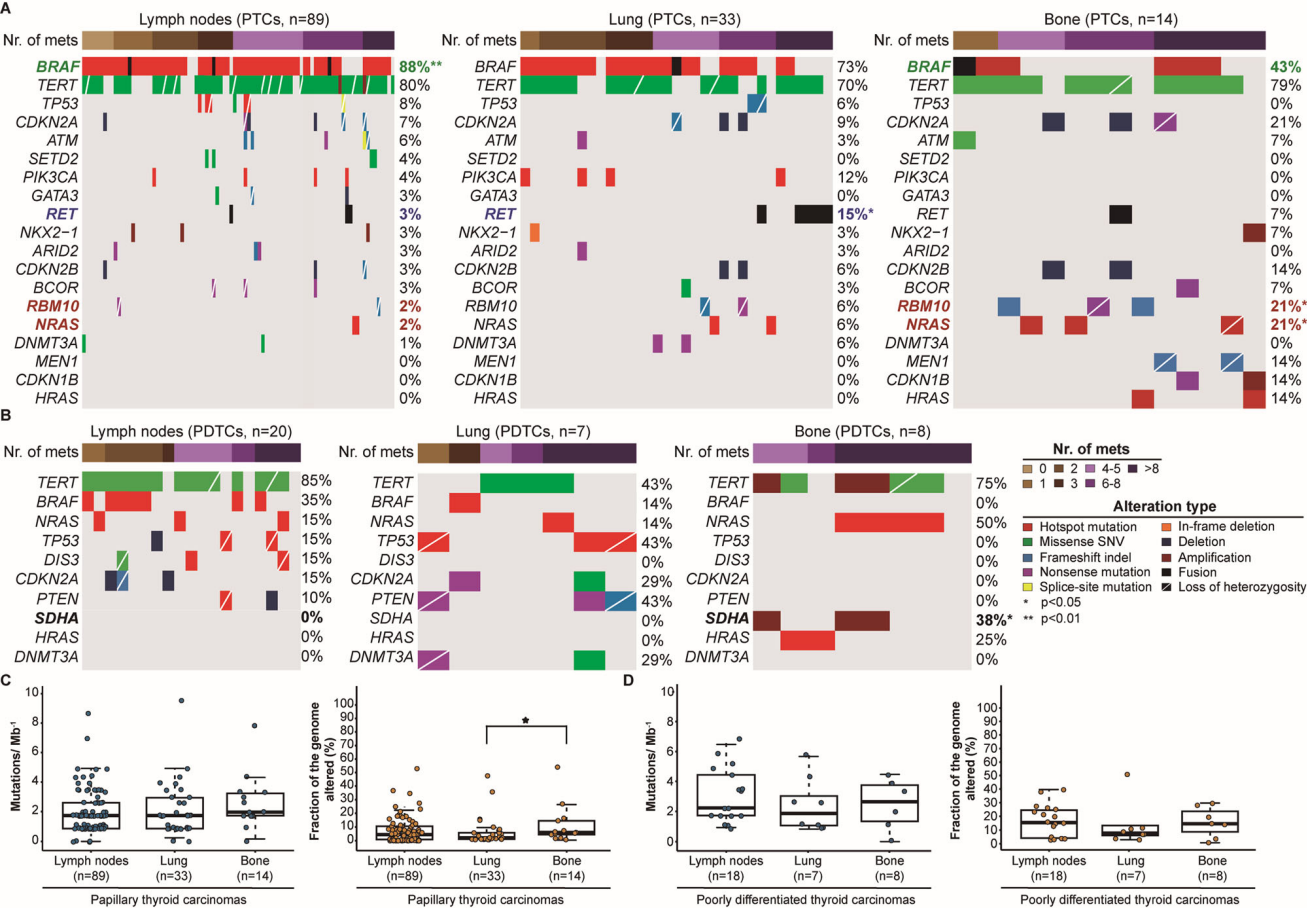
Genetic features of metastatic papillary thyroid carcinomas and poorly differentiated thyroid carcinomas. Comparisons between lymph node metastases (LNMs) and distant metastases (DMs) from papillary thyroid carcinomas for (**A**) recurrent somatic alterations and (**C**) tumor mutational burden (TMB) and fraction of the genome altered (FGA). Comparisons between LNMs and DMs from poorly differentiated thyroid carcinomas for (**B**) recurrent somatic alterations and (**D**) TMB and FGA. The number of metastases and alteration types are color-coded according to the legend. Statistical significance was evaluated in **A** and **B**, using Fisher’s exact test, and in **C** and **D** using Mann–Whitney U test. PTCs papillary thyroid carcinomas, PDTCs poorly differentiated thyroid carcinomas

With the purpose of characterizing the genetic repertoire of metastatic PTC and PDTC patients (Fig. 1A–D), we first assessed the genetic differences between the LNMs and the different DMs from PTC patients. We observed that the frequency of *BRAF* hotspot mutations gradually decreased from the LNMs to the lung metastases and to the bone metastases, with the frequency of *BRAF* mutations in the latter being significantly lower than in the LNMs (43% vs 88%, *p* < 0.01). In addition, a significantly higher frequency of *RMB10* and *NRAS* pathogenic mutations was found in the bone metastases than in the LNMs (21% vs 3%, *p* < 0.05 for both genes) (Fig. 1A). The lung metastases were found to harbor a significantly higher frequency of *RET* translocations when compared to the LNMs (15% vs 3%, *p* < 0.05) (Fig. 1A). Among the LNMs and the different DMs, the bone metastases had the highest TMB (median: 1.96, range: 0–7.8), although no statistical significance was reached (Fig. 1C). Nonetheless, we noticed a significantly higher FGA in the bone metastases than the FGA of the lung metastases (5.6% vs 1.3%, *p* < 0.05) (Fig. 1C).

The genetic profile of the LNMs from PTC patients harboring 4 or more DMs revealed a significantly higher frequency of *TERT* promoter mutations than the LNMs from PTC patients harboring less than 4 DMs (96% vs 65%, *p* < 0.001), and a higher frequency of *ATM* pathogenic mutations, being such difference borderline significant (11% vs 0%, *p* = 0.058) (Supplementary Fig. S1A). In addition, the frequency of *RET* translocations, although not statistically significant, was substantially higher in the lung metastases from PTC patients harboring 4 or more DMs than the lung metastases from PTC patients with less than 4 DMs (24% vs 6%, *p* > 0.05) (Supplementary Fig. S1B).

### Bone metastases from PDTCs are enriched in *SDHA* and *TERT* gene amplifications

The genetic repertoire of the metastasis from PDTC patients revealed an enrichment of copy number amplifications affecting *SDHA* and *TERT* genes in the bone metastases when compared to the LNMs, being the frequency of the former significantly higher (38% vs 0%, *p* < 0.05) (Fig. 1B). Although not statistically significant, we noticed a considerably lower frequency of *TERT* promoter mutations in the lung metastases than in the LNMs (43% vs 85%, *p* > 0.05) and bone metastases (43% vs 75%, *p* > 0.05) (Fig. 1B). We also observed the absence of *BRAF* hotspot mutations in the bone metastases, and a substantial higher frequency of *TP53* pathogenic mutations in the lung metastases when compared to the LNMs (43% vs 15%, *p* > 0.05) and bone metastases (43% vs 0%, *p* > 0.05) (Fig. 1B). The bone metastases had the highest TMB (median: 3.3, range 0–3.9) when compared to the LNMs and lung metastases, while the LNMs had the highest FGA (median: 15.4%, range: 2.8%-39.5%) (Fig. 1D). Although no statistical significance was reached, a lower frequency of *BRAF* hotspot mutations was found in the LNMs from PDTC patients with 4 or more DMs when compared to the LNMs from PDTC patients with less than 4 DMs (18% vs 57%, *p* > 0.05) (Supplementary Fig. S1C).

## Discussion

Considering the clinical difficulties in treating PTCs and PDTCs harboring DMs, a precise description of the genetic repertoire of these advanced TCs is demanded. Here, we retrieved and analyzed the molecular and clinical features of the LNMs and DMs from PTC and PDTC patients using a subset of the series taken from cBioportal and recently reported by our group [2]. The genetic repertoire of the metastases from PTCs revealed a significantly higher frequency of *BRAF* hotspot mutations affecting the LNMs than the bone metastases. We and others have previously observed an enrichment of these hotspot mutations in the LNMs when compared to the DMs [2, 9, 10] and suggested that other driving events could be more likely linked to the DMs. In this study, we observed an enrichment of *NRAS* and *RMB10* pathogenic mutations in the bone metastases, and an enrichment of *RET* translocations affecting the lung metastases. Despite the scarcity of genetic studies regarding these lesions, a case report involving a patient with aggressive differentiated carcinoma with multiple DMs (including bone metastases) revealed the presence of the *NRAS Q61R* hotspot mutation in all the DMs, and concluded that *NRAS*-mutant thyroid carcinoma exhibited a high risk of developing bone metastases [11]. Of note, and akin to what was previously observed in the DMs from PDTCs [2], *BRAF* hotspot mutations do not seem to be a driver of the bone metastasis from either PTC or PDTC patients. In addition, and considering the presence of *RET* rearrangements already identified in 1–3% of non-small cell lung cancers [12], and the poor overall survival already demonstrated in advanced PTC patients harboring such translocations [2], we re-highlight the need of using highly specific small-molecule *RET* inhibitors [13] in patients with *RET*-rearranged advanced TCs.

While the presence of high frequencies of *TERT* promoter mutations in metastatic PTCs was already demonstrated to be associated with tumor aggressiveness [14–16], the enrichment of *ATM* pathogenic mutations in the LNMs of PTC patients with 4 or more DMs observed in this study is noteworthy. The presence of mutations affecting such DNA repair gene was also demonstrated to affect 4–7% of advanced PTCs [17, 18], thus highlighting the need of using next-generation sequencing analysis for the detection of low-level gene mutations in advanced TCs, and for a proper therapeutic consideration of these patients.

In metastatic PDTC patients, an enrichment of *SDHA* and *TERT* gene amplifications were also observed in the bone metastases. Although not commonly observed in TCs, *SDHA* amplifications were recently observed in endometrial carcinomas (ECs), and was also found to be co-amplified with *TERT* in 61% of *TERT* amplified ECs [19]. In addition, SDHA overexpression was demonstrated to improve survival of ovarian tumor cells and their ability to generate colonies in anchorage-independent conditions, thus favoring the spreading of these tumor cells [20]. Interestingly, in vitro and in vivo studies have demonstrated a profound anti-tumor efficacy and selectivity of shikonin (an anti-metabolic compound) towards SDHA overexpressing ovarian tumor cells, superior to that observed with chemotherapy [20]. Although our cohort is rather small, the presence of a subset of PDTCs affected with *SDHA* amplifications could be successfully targetable, offering a potential new treatment strategy for these patients. Nonetheless, we cannot exclude the presence of a co-amplification with *TERT*, since both genes are very close in chromosome 5.

In conclusion, we demonstrate that the metastases from PTCs and PDTCs harbor clinically relevant alterations associated to distinct body locations, such as *NRAS* and *RBM10* mutations, *RET* translocations and *SDHA* amplifications, of which some of them may be explored therapeutically.

## Supplementary information

Supplementary Information
