# Supplementary material for: Genomic profiling of lymph node and distant metastases from papillary and poorly differentiated thyroid carcinomas
Source: Endocrine. 2024 Jul 19;86(2):505–9. doi: 10.1007/s12020-024-03968-0 (PMC11489207; doi:10.1007/s12020-024-03968-0)
Supplement: Supplementary file 1 — Supplementary Information [file 12020_2024_3968_MOESM1_ESM.docx]

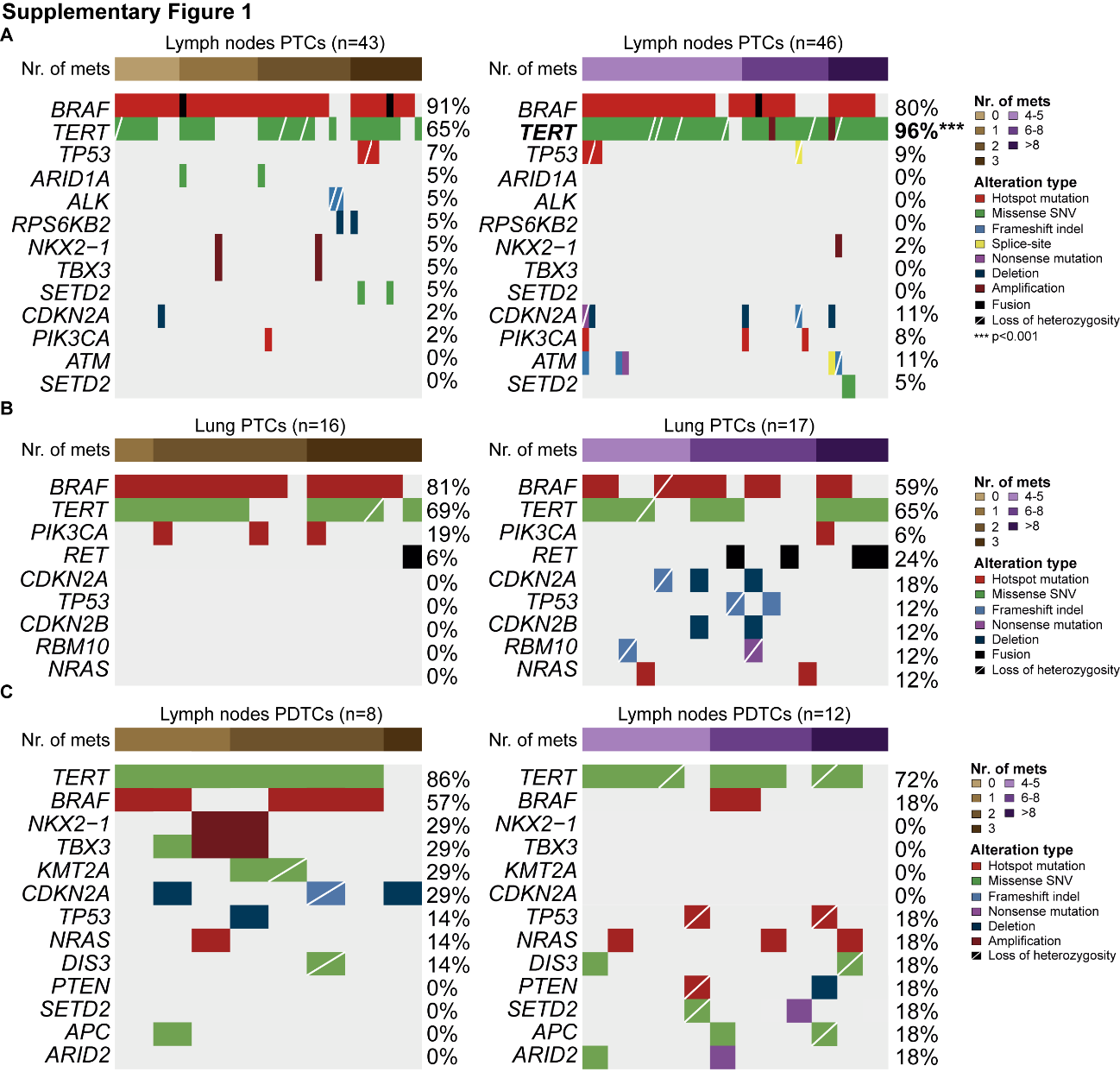


**Supplementary Figure 1: Genetic features of lymph node metastases from papillary thyroid carcinomas (PTCs) and poorly differentiated thyroid carcinomas, and genetic features of lung metastases from PTCs.** Comparisons between lymph node metastases (LNMs) and lung metastases from papillary thyroid carcinoma patients stratified according to the number of distant metastases (DMs) for (**A**, **B**) recurrent somatic alterations. Comparisons between LNMs from poorly differentiated thyroid carcinoma patients stratified according to the number of DMs for (**C**) recurrent somatic alterations. The number of metastases and alteration types are color-coded according to the legend. Statistical significance was evaluated in (**A**), (**B**) and (**C**) using Fisher’s exact test. PTCs, papillary thyroid carcinomas; PDTCs, poorly differentiated thyroid carcinomas.
